# Evolutionary Potential of a Duplicated Repressor-Operator Pair: Simulating Pathways Using Mutation Data

**DOI:** 10.1371/journal.pcbi.0020058

**Published:** 2006-05-26

**Authors:** Frank J Poelwijk, Daniel J Kiviet, Sander J Tans

**Affiliations:** FOM Institute for Atomic and Molecular Physics (AMOLF), Amsterdam, Netherlands; MRC Laboratory of Molecular Biology, United Kingdom

## Abstract

Ample evidence has accumulated for the evolutionary importance of duplication events. However, little is known about the ensuing step-by-step divergence process and the selective conditions that allow it to progress. Here we present a computational study on the divergence of two repressors after duplication. A central feature of our approach is that intermediate phenotypes can be quantified through the use of in vivo measured repression strengths of *Escherichia coli lac* mutants. Evolutionary pathways are constructed by multiple rounds of single base pair substitutions and selection for tight and independent binding. Our analysis indicates that when a duplicated repressor co-diverges together with its binding site, the fitness landscape allows funneling to a new regulatory interaction with early increases in fitness. We find that neutral mutations do not play an essential role, which is important for substantial divergence probabilities. By varying the selective pressure we can pinpoint the necessary ingredients for the observed divergence. Our findings underscore the importance of coevolutionary mechanisms in regulatory networks, and should be relevant for the evolution of protein-DNA as well as protein-protein interactions.

## Synopsis

The evolution of a new trait critically depends on the existence of a path of viable intermediates. Generally speaking, fitness decreasing steps in this path hamper evolution, whereas fitness increasing steps accelerate it. Unfortunately, intermediates are hard to catch in action since they occur only transiently, which is why they have largely been neglected in evolutionary studies.

The novelty of this study is that intermediate phenotypes can be predicted using published measurements of Escherichia coli mutants. Using this approach, the evolution of a small genetic network is simulated by computer. Following the duplication of one of its components, a new protein-DNA interaction develops via the accumulation of point mutations and selection. The resulting paths reveal a high potential to obtain a new regulatory interaction, in which neutral drift plays an almost negligible role. This study provides a mechanistic rationale for why such rapid divergence can occur and under which minimal selective conditions. In addition it yields a quantitative prediction for the minimum number of essential mutations.

## Introduction

Initially put forward by Stevens in 1951 [1] and later advocated by Ohno in his seminal work [2], gene duplication followed by functional divergence is now seen as a general mechanism for acquiring new functions [3]. Also, regulatory networks are thought to be shaped significantly by genetic duplication [4]. For instance, sequence analysis of transcription factor families points to various historical duplication events [5,6]. However, very little is known about the subsequent mutational divergence pathways or about the corresponding stepwise phenotypical changes that are subject to selection. While these issues have not yet been explored experimentally, related generic aspects of mutational plasticity have been addressed theoretically [7–11]. However, a central obstacle in studying mutational pathways through computer simulations remains the unknown relation between the sequence and binding affinity, for which, in general, a rather abstract mapping has to be assumed. To describe the formation of a new regulatory interaction after a duplication event, which is our current aim, such an abstract approach would be particularly speculative.

Here we reason that many characteristics of the adaptation of real protein-DNA contacts are hidden in the extensive body of mutational data that has been accumulated over many years (e.g., [12–14] for the *Escherichia coli lac* system). These measured repression values can be used as fitness landscapes, in which pathways can be explored by computing consecutive rounds of single base pair substitutions and selection. Here we develop this approach to study the divergence of duplicate repressors and their binding sites. More specifically, we focus on the creation of a new and unique protein-DNA recognition, starting from two identical repressors and two identical operators. We consider selective conditions that favor the evolution toward independent regulation. Interestingly, such regulatory divergence is inherently a coevolutionary process, where repressors and operators must be optimized in a coordinated fashion.

The mere presence of a selective pressure is clearly not a sufficient condition to achieve a new function. Rather, the evolutionary potential and limitations can be seen as governed by the shape of the actual fitness landscape and the evolutionary search within it. Studying these intrinsic limitations to divergence represents the overall aim of this work. Many open questions arise when considering the formation of a new protein-DNA interaction, which may be viewed as the construction of a new lock and uniquely matching key. For instance, how should the protein be modified step-by-step to recognize a new DNA-binding site that also does not yet exist, or vice versa? One would expect that complementary mutations need to occur in the protein and DNA-binding site. Does this mean that temporary losses in fitness must be endured when taking single-mutation steps? And, how many mutations must minimally accumulate before a noticeable new recognition is obtained on which selection can act? The latter is an important point: mutations conferring a selective advantage spread more readily through a population [15], resulting in a drastic increase of the divergence probability. These questions are addressed by exhaustively searching the landscape for optimal pathways, as well as by complementary population dynamics simulations.

Previously it has been shown that *lac* repressor mutants indeed exist that can bind exclusively to mutant *lac* operators [14]. Our simulations reveal that a duplicated repressor-operator pair can readily evolve to achieve such independence of binding, while monotonously increasing its fitness in a step-by-step process. Moreover, simply following the fittest mutants does predominantly guide the system to the desired global optimum, which indicates funnel-like features in the fitness landscape. A detailed analysis of the subsequent network changes indicates a generic sequence of events, of which we study the underlying mechanisms by varying the applied selective pressure. Next, we show that the trajectories we find in the optimal pathway simulations are not rare exceptions, since similar trajectories are followed using a probabilistic scheme for accepting a mutation. The results further suggest the feasibility of studying regulatory divergence in laboratory evolution experiments, and finally we make a comparison to alternative models for the creation of new regulatory interactions.

## Results

### Selective Pressure and Fitness Landscape

**Figure 1:**
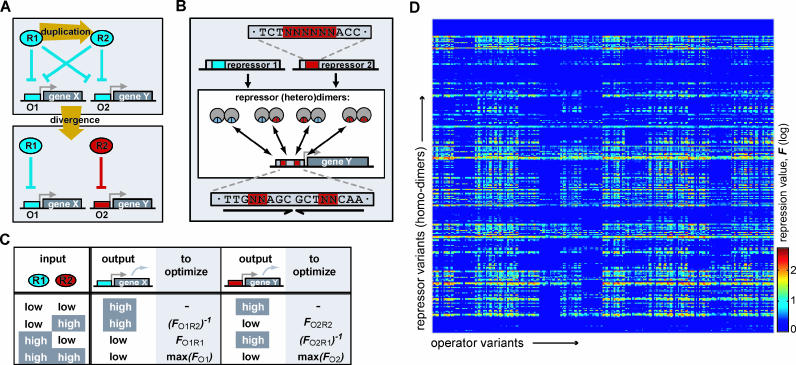
Divergence Process, Fitness Criterion, and Mutational Dataset of Repression Values (A) Diagram of the studied divergence process: after a duplication event, a new regulatory interaction can be formed by mutating the two operators, O1 and O2, and two repressors, R1 and R2. (B) Duplication and divergence yields heterodimers, which can all bind to the operator. The (initially symmetric) operators and repressors are based on the *lac* sequence, as indicated. Base pairs that are key to altering specificity (colored red and blue) can be mutated to arbitrary sequence. (C) The selective pressure for independent regulation follows from four input conditions that contribute to the total fitness. When, e.g., R1 is high and R2 low, this implies that X should be low and Y high. Out of all interaction parameters of the network, in this case only *F*_O1R1_ and (*F*_O2R1_)^−1^ are relevant and need to be optimized. When R1 and R2 are high, both X and Y should be low, regardless of which repressor-dimer causes repression. Therefore max(*F*_O1_) (the strongest interaction with O1 by either homodimers of R1 or R2 or by the heterodimer of R1 and R2) and max(*F*_O2_) need to be be optimized. When both R1 and R2 are low, no parameters need to be optimized. (D) Resulting repression value landscape, showing repression values based on actual measurements of mutants.

We consider an ecological situation where natural selection would favor independent regulation of two genes X and Y. Regulation is not independent in the initial symmetric network with duplicated components (see Figure 1): X and Y have two identical upstream binding sites (O1 and O2), which bind two identical repressors (R1 and R2) equally strongly. Such a situation will, for instance, arise upon duplication of a repressor that regulates two or more genes. Note that this selective pressure, of course, is not a general outcome of a repressor duplication. A duplication event may arise in the context of a different functional pressure, which could direct the evolution toward a different topological motif [16]. Most often, selective pressures for a new function will be absent, in which case silencing of one of the duplicates is the most probable outcome [3,17]. However, the rare cases where a selective pressure is present are crucial to developing new functions.

We aimed to define a transparent selection pressure for the divergence of these regulatory interactions. Attributing a fitness value to a network function is non-trivial: unlike for an enzymatic function, network fitness cannot be captured in a single biochemical parameter. Here we propose to assign a fitness value based on the desired input-output relation of the network (see Figure 1A and 1C). For simplicity, only two concentration levels (high and low) of input and output protein are considered, resulting in four possible input conditions. For each of these input conditions, it follows straightforwardly which repressor-operator interactions should be maximized and which must be minimized. The interaction strength between operator Oi and repressor homo-dimer Rj is expressed by repression values (*F*_OiRj_). This value represents the expression level of a downstream gene in the unrepressed condition divided by the repressed condition and it is obtained directly from measured data (see below and Materials and Methods). Taking the fitness to scale linearly with the repression values, the fitness of the complete network is denoted by the product of all optimization factors:


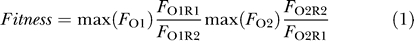


In this expression max(*F*_Oi_) denotes the repression value of the strongest interaction with Oi, either by homodimers of R1 or R2 or the hetero-dimer composed of R1 and R2 (see Figure 1 and Materials and Methods).

The fitness definition comes down to a minimum set of two demands for regulatory binding: each operator must bind a repressor tightly (max(*F*_O1_) and max(*F*_O2_) should be large) but also exclusively (*F*_O1R1_ */F*_O1R2_ and *F*_O2R2_ */F*_O2R1_ should be large). Prior to divergence the first demand is already met, but the latter is not. The challenge during divergence is therefore to improve binding exclusivity, while maintaining tight binding. Tight and exclusive binding is a core functionality of most regulatory systems, and most pairs of existing transcription factors must therefore score well on the employed fitness definition. Take for instance the LacI and RafR repressors, which regulate enzymes required for growth on lactose and raffinose, respectively. If operator binding would not be tight in the absence of lactose and raffinose, the wasteful expression of the downstream metabolic enzymes would lead to sub-optimal growth speeds [18,19]. If RafR would also bind to the *lac* operator (and thus bind non-exclusively), the effect on growth speed would also be negative since the mere absence of raffinose would then lead to insufficient β-galactosidase for high lactose concentrations.

One therefore expects a conservative selective pressure that minimally includes binding tightness and exclusiveness, to keep the *lac* and *raf* regulation intact. Important here is that the *lac* and *raf* repressors are in fact related: their origin has been traced to duplication events from a common ancestor [6]. If a conservative pressure keeps their function intact now, it seems a good candidate for the initial divergence pressure as well. Full divergence to the current *lac* and *raf* systems clearly involves many additional developments after duplication. For instance, the divergence of ligand-binding properties [20] might have occurred prior to operator-binding divergence. While these considerations put additional constraints on the entire divergence process, they do not alter the particular operator-binding divergence studied here.

A remaining question still is how the various demands should be weighed in the total fitness. That choice is clearly not general: it will strongly depend on the operons in question and on the changing cell environment. For example, if active RafR is present more than half of the time, then its cross-interaction with the *lac* operator would be comparatively more harmful because it lasts longer. In order to give a uniform presentation we weighed the factors of the four input states equally, which would correspond to an equal contribution of these phases to the overall fitness. However, weighing the factors unequally (e.g., by increasing the power of the tight operator binding, or the cross- interaction factors from 1 to 2) did not alter the main conclusions.

### Mutation Data and Pathway Simulations

In our simulations, the strength of a mutant repressor-operator interaction (as expressed by the repression value *F*), is assigned using data from mutational analysis [14]. In these experiments, repression values have been determined in vivo from the repressed and unrepressed expression levels of a *lacZ* gene, controlled by a mutant *lac* operator and mutant *lac* repressor (see Materials and Methods). Obviously not all possible *lac* mutants have been constructed. Therefore, a potentially significant limitation of our simulations is the restricted number of base pairs that can be mutated in silico and linked to experimental data. At the same time however, while the tightness of DNA binding is the result of the integral protein architecture, surprisingly few base pairs (ten in total) have been found to be important for altering binding specificity [14] (see Figure 1B). Focusing on these key base pairs is therefore reasonable for the minimal paths that we are interested in here. Using measurements on 1,286 mutants, repression values of *all* variants in these key base pairs could convincingly be determined [13,14,21]. These variants thus include all multiple mutants in both the repressor and the operator. Repression values of heterodimers and asymmetric operators are calculated using an additive contribution of the repressor monomers to the dimer-DNA binding [22] (see Materials and Methods). In total, about 1 × 10^7^ possible repressor-operator combinations are obtained (see Figure 1D for the homodimer variants).

Every mutational path starts with the duplicated sequence of a tight binding repressor-operator combination (repression value ≥ 100). These possible starting sequences obviously include wild-type *lac,* but also e.g., the *gal* and *ebg* systems, which are part of the same family of repressors. Their high measured repression values are rather remarkable because the rest of the *gal, ebg,* and *lac* sequences have in fact diverged considerably. These observations further indicate that the key base pairs play the central role in specific recognition.

The aim of the simulation method (see Materials and Methods for details) has been to reveal the intrinsic possibilities for the divergence of repressor-operator binding, given the measured data and the constraints of single base pair substitutions and no fitness decreases. For this purpose, we search the landscape for optimal paths and study what their limitations and potential are. To trace these optimal paths, all mutants with a single base pair substitution with respect to their parents are evaluated based on the fitness described above, and the best performers are selected for the next round. The number of selected mutants *L* is varied to assess its effect. We also question whether these optimal paths are not just rare cases, by comparing them with pathways generated by a different simulation method, where a random mutation is accepted with a probability that depends on its associated fitness increase [23] (see Protocol S1).

### Short Co-Divergence Pathways

**Figure 2:**
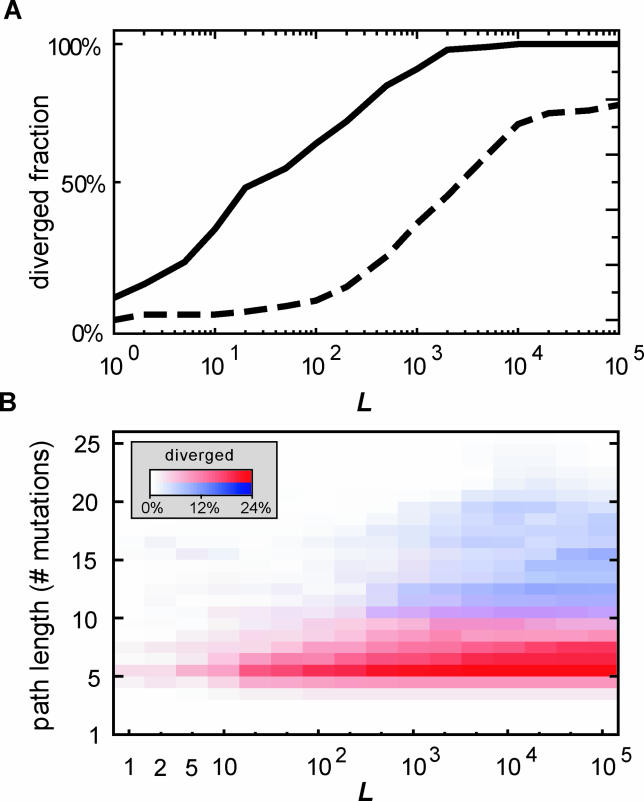
Divergence Success Ratio and Path Length Distributions (A) Fraction of starting sequences (numbering 132 in total) that successfully diverge, as a function of the number of networks carried to the next round *(L).* Dashed line, idem, but with the additional requirement of continued tight binding (*F* ≥ 100) for both repressors. (B) Distribution of path lengths until divergence. Red color map, optimal co-divergence pathways. Blue color map, pathways with the additional requirement of *F* ≥ 100 for both repressors. Note that a vertical summation of the color maps yields the lines in (A).

The simulations show that paths to independent recognition are readily found. Even when only the best network is carried to the next round (*L* = 1), which implies always following the steepest ascent in fitness, some starting sequences can evolve to the highest fitness in the sequence space. In these networks, both repressors bind tightly to one operator (*F*_O1R1_ = 520 and *F*_O2R2_ = 200, respectively), while not at all to the other (*F*_O1R2_ = 1, *F*_O2R1_ = 1). We considered paths to be successful when the fitness value is within an order of magnitude of the highest fitness in the landscape, which is a strict criterion given the fact that the fitness parameter is a product of six factors. The diverged fraction increases for higher *L* (Figure 2A, solid line), which is expected since it allows alternative paths to be explored. More surprising is that successful trajectories can eventually be found from all starting points, but note that paths that can only be followed for higher *L* are increasingly less probable because they imply more (near) neutral mutations.

**Figure 3:**
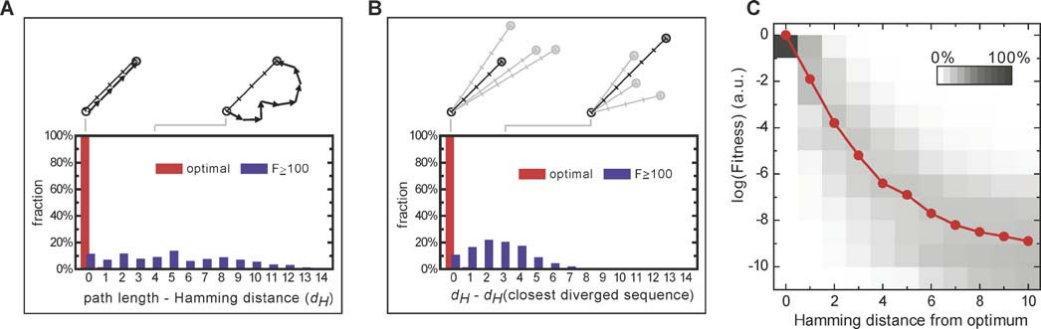
Analysis of Pathway Detours and Local Environment of Fitness Optima (A) Histogram showing the number of detour mutations of the divergence pathways. The Hamming distance *d_H_* of two sequences is defined as the number of positions at which they have different base pairs. Paths that are longer than *d_H_* arrive at an optimum after a detour. (B) Histogram of the Hamming distance between the optimum that is found and the closest optimum. If this measure is zero, a path leads to the closest optimum. (C) Median fitness value as a function of the Hamming distance from a global optimum (solid line). Gray levels indicate the spread of the fitness values.

**Figure 4:**
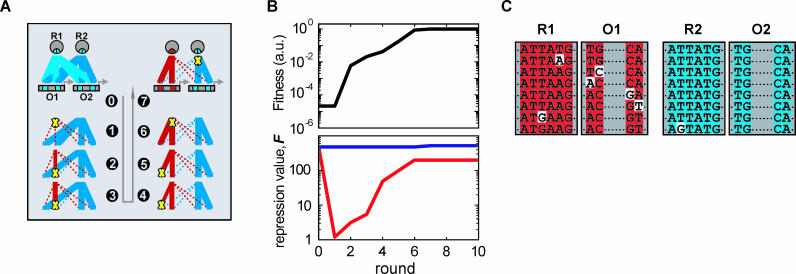
Typical Divergence Pathway: Network Changes, Fitness, and Sequence (A) Evolving interaction network, where line thickness denotes binding strength between repressor monomer and operator-half. Dotted lines denote negligible repression. Yellow crosses indicate repressor and operator mutations, which are positioned at the top and bottom of the interaction lines respectively. (B) Fitness trajectory (black) and corresponding repression of each repressor on its operator (red and blue). Fitness is normalized to the maximum value (~1 ×10^10^). (C) Sequences for each round. Mutated positions are colored white.

Most optimal paths are rather short: 70% require just five to nine mutations for *L* = 20 (Figure 2B). The systems almost exclusively find the nearest diverged state in sequence space (Figure 3B) and do so without taking any detours (Figure 3A). Notably, despite the fact that the starting points lie in very different areas of the sequence space, a generic sequence of network changes is generally observed (see Figure 4 for an example). First of all, one repressor-operator combination remains unchanged, except at the very end, as the other diverges away. This is an example of asymmetric divergence due to positive selection, as has also been found in phylogenetic analysis of duplicate genes in eukaryotes [24]. A striking general feature of the pathways is an early reduction in the binding strength of the diverging repressor, brought about by a single base pair substitution (Figure 4B, red trace). Such a mutation would be unfavorable for a single repressor-operator pair, but here it can be fitness neutral, partly because the unchanged duplicate repressor ensures a continued repression. At this specific point the diverging repressor is freed from functional constraints and therefore most vulnerable to degenerative mutations resulting in silencing of the gene. The probability of silencing is reduced however, because already at the second mutation and onward, new and unique protein-DNA recognition can be built up. At the sequence level, this phase is characterized by transient asymmetries. The operator must go through non-palindromic sequences because it can only receive one mutation at a time. Heterodimers are the best binders in this phase because of their ability to mirror the non-palindromic operator sequences. Eventually all successful trajectories recover palindromic operators, even as the selective pressure does not explicitly specify this. With all dimer varieties present, a homodimer is available and now binds most tightly to the palindromic operator.

In order to obtain a better insight in the essential ingredients for the observed evolvability, various additional simulations were performed. For instance, we were triggered by the recurrent early knockout of one of the repressors, which is one of the most noticeable features of the mutational pathways. To test for the importance of this step, both repressor-operator pairs were required to maintain a significant repression (*F*_O1R1_ ≥ 100 and *F*_O2R2_ ≥ 100). Divergence is indeed significantly frustrated by these conditions (Figure 2A, hatched line). The amount of selected mutants needs to be two orders of magnitude larger (*L* > 1,000) for half of the starting sequences to diverge. The saturation of the diverged fraction for very high *L,* where prolonged neutral drift is allowed, indicates that for 22% of the starting sequences no pathways exist. Moreover, in contrast to the optimal paths, the nearest diverged state in the landscape is generally *not* found, and the paths contain significant detours (Figure 3). The same is seen from the increased path length: 70% of the paths take 11–21 mutations (Figure 2B). These paths lack a recurring mutation pattern as observed for the optimal paths and instead show a large variation in the sequence of events. Both repressors and operators are significantly mutated, and the fitness increases slowly or is neutral over multiple rounds (see Figure S1 for an example).

Another defining feature of duplicated transcription factors is the heterodimerization of transcription factor monomers. It is not a priori evident whether this constraint on the network topology either promotes or hampers divergence. To assess its effect, simulations were performed where heterodimers are not able to form (unpublished data). The results indicated a surprisingly limited effect on the divergence. The paths do initially show a slower fitness increase, but the path length does not appear much affected, nor the success rate of divergence. The other simulation variations we conducted (with unequally weighted factors in the fitness definition), did not qualitatively alter the main divergence features, such as substantial divergence success without fitness decreases, short paths, and an early repression dip, indicating the robustness of our results.

## Discussion

### Duplication and Coevolutionary Divergence

We obtain a first view on a fitness landscape for regulatory divergence that is based on actual measured data. We show that the landscape allows evolutionary paths toward independent repressor-operator interactions, exhibiting a step-by-step increasing fitness, starting as early as the first or second mutation. Since the possibility of following such paths critically depends on molecular properties, the use of empirical data is essential for such claims. One could also have imagined fitness landscapes where paths to diverged networks do not exist, or where they are very long, involving large detours. Our results contrast with the notion that a number of neutral or even deleterious mutations have to accumulate before a new function can develop (see for a discussion e.g., [25]). Having beneficial mutations available early on is important, since it greatly enhances divergence probabilities [15]. A lack of early selection would result in much higher probabilities of silencing of one of the duplicates by the accumulation of mutations [3,17].

While the presented systematic search for optimal pathways is useful in revealing necessary conditions for divergence, one may wonder whether paths are not very different in a probability-based fixation process that typifies natural evolution. However, we found that population genetics simulations reveal the same pathway characteristics: a significant fraction of paths are successful with monotonous fitness increases, one repression dip early on, and few neutral mutations are present (see Protocol S1 and Figure S2).

The coevolutionary search for a new and independent recognition, which is relevant for both protein-DNA and protein–protein interactions, comprises fundamental differences with often-considered evolution of ligand-binding and enzymatic activity [26–28]. While in the latter cases the new evolutionary target is fixed, here it is open-ended: as with locks and keys, many possible combinations are unique matches, and each of those is equally suitable. This large degree of freedom allows the system to choose the solution that is most accessible. Another difference with fixed-target evolution lies in the selective pressure. Binding is already tight to both operators at the start of the coevolutionary scenario, so the initial pressure to change, in fact comes from benefits of *not* binding another operator. This pressure for unique recognition is characteristic for regulatory interactions but plays much less a role in developing other functions such as enzymatic activity. These characteristics of a coevolutionary mechanism, together with the remarkable plasticity of protein-DNA interactions result in a highly evolvable system.

### Fitness Landscape Funnels

The diversity of molecular architectures is not only constrained by their *inherent* physico-chemical limitations, but also by the existence of viable evolutionary routes that shape them. For instance, in a population of bacteria there is only a small probability that an advantageous function emerges *if* a temporary fitness decrease is required first. Put differently, the shape of the fitness landscape is critical, and one can readily imagine fitness landscapes where the optima are very difficult to reach. Upon first inspection, the measured landscape we consider indeed contains many potential frustration sources: over 99% of all optima in the landscape are in fact below our divergence criterion. Such local optima represent traps in which the system gets permanently stuck once it encounters one. However, the results show that the system is still guided in the right direction to (near) global optima, which indicates that the fitness landscape contains funnel-like features. Moreover, the optimal paths contain negligible detours (Figure 3A) and lead to the nearest optimum (Figure 3B), showing that the funneling is efficient and not constrained by ruggedness. A funnel-like organization of the landscape is also supported by the monotonous fitness increases of the probabilistic pathways (Figure S2C), as well as by the smooth fitness decrease when stepping away from a global optimum (Figure 3C).

The underlying causes for funnels in the fitness landscape may be found at two levels. The first level is that of a single repressor-operator interaction. The surface smoothness that is needed for the funnels may be partly understood from the reported additive contributions of the *lac* amino acids to the binding energy. In mathematical models, additive interactions have been shown to yield smoother fitness surfaces because they can be optimized independently [7].

At a higher level, features of the network topology shape the landscape surface and divergence potential. We found that the tightly interconnected topology, as present after the duplication, does not frustrate divergence but instead promotes it. In contrast to an isolated repressor-operator pair, where a drop in the binding strength decreases the fitness, the same mutation can be neutral in the interconnected topology. Compensation for the decrease in binding strength can be attributed to two features of the topology. First, there is the characteristic pressure to *not* bind the rival operator: when a mutation decreases an interaction that should be maximized, this negative effect on the fitness is partly balanced by the decrease of an unwanted cross-interaction. A second mechanism is a coevolutionary twist on Ohno's original idea, in which one repressor-operator pair can search for a new recognition, while the other repressor maintains repression on both operators in the very early stages. As we have observed that a drop in the binding strength is necessary for efficient divergence, the ability to compensate for its negative contribution to the fitness is crucial for funneling.

The evolutionary fate of redundant genes has previously been studied primarily using sequence analysis [3,29]. By using a different dataset and approach, our simulations strengthen recent evidence for a more rapid fixation of mutations in redundant genes [29] (termed “accelerated evolution”). Our analysis enables a next step in our understanding of this important process: It provides a mechanistic rationale for why such a rapid divergence can indeed occur, in terms of minimal selective conditions bacteria must experience, in combination with independently measured plasticity of protein-DNA interactions. Furthermore it yields a quantitative prediction for the minimum number of essential mutations to achieve divergence.

### Suggested Experiments

Our results show divergence to be possible with monotonic increasing fitness, which hints at the feasibility of monitoring similar processes in experiments. It has recently been shown that the serial dilution assay, as pioneered by Lenski and coworkers [30], can be employed to adapt bacterial strains to a new condition within weeks [19,31]. Similarly, one could attempt to evolve a duplicate *lac* repressor/operator copy towards the independent regulation of a second operon. However, this more complex assay does require key modifications: (1) growth and selection of the mutants should occur in alternating media, in analogy to our discussion of multiple input conditions, and (2) a starting network must be engineered that satisfies the conditions for DNA-binding divergence: a duplicate repressor/operator and a selective pressure for tight and independent binding.

In practice, one could place the *lac* operator upstream of the raffinose utilization operon, and construct a *lacI* duplicate that is sensitive to raffinose. This initial situation is now similar to our simulations: two *lac* repressors bind to the two *lac* operators. The employed fitness definition is also suitable: (1) in media where the two metabolites are both low (supplemented e.g., by another carbon source), the metabolic enzymes should not be expressed. The resulting optimal growth is well represented by positive contributions to the overall fitness by high values for tight binding. (2) When just one metabolite is present, one screens for exclusive binding. In a medium without lactose the lactose-sensitive repressor shuts both operons down if binding is still non-exclusive. Upon mutations that allow this repressor to bind exclusively to the operator of the lactose operon, raffinose metabolic enzymes would be expressed. The resulting faster growth due to raffinose utilization thus correlates well with higher values for exclusive binding. The pressure for a correct behavior under multiple conditions prevents the fixation of trivial solutions that would just work under one condition.

### Other Network Growth Scenarios

For biological regulatory networks to grow, not only new components are required, but also new and independent interactions. Next to the coevolutionary duplication-divergence scenario for network growth, alternative models for the creation of new regulatory interactions have been proposed. In the first alternative, a new operator must emerge upstream of the regulated gene in an effectively random DNA sequence [32]. This scenario has mainly been considered for eukaryotes with large upstream regulatory regions and short binding sites. For longer operators in prokaryotes, this scenario requires many neutral mutations before improvements can be selected for (see Protocol S2), which represents a major evolutionary obstacle.

Another possible source for new regulatory interactions is lateral gene transfer, which is thought to be the source of many paralogs found in prokaryotes [33]. In this scenario divergence would occur while two genes each reside in different organismal lineages (essentially being orthologs at that stage) and each experiencing different selective constraints. Lateral gene transfer unites the diverged genes, resulting in immediate contributions to fitness by both homologous genes. Although examples of this scenario have been found for enzymes [34], transcription factor-operator interactions are a special case, as there is no obvious internal or external selection pressure for their interaction to diverge by itself. Our results illustrate the feasibility of coevolutionary divergence of two transcription factors within a single organismal lineage. These findings are supported by the lack of evidence for horizontal transfer of the *lac* system in E. coli [35]. However, this is not to say that lateral gene transfer and duplication-divergence are mutually exclusive. Summarizing, the coevolutionary divergence studied here differs from alternative models of network growth by providing both a high probability of selective advantageous point mutations and a rationale for a divergence pressure.

Finally, it is of interest to consider different selective pressures within the same duplication scenario. While the pressure for independent regulation seems to be a dominant one, as evidenced by the many independent transcription factors that are paralogs, duplications also have yielded other network motifs. An interesting example is the UxuR/ExuR pair of repressors. Like the case studied in this paper, they have originated by duplication and share two operators (see Protocol S3). However, they seem to have diverged under a different selective pressure, since their cross interaction was not eliminated, but instead has been retained, forming a so-called *bi-fan* motif [16].

This work describes how regulatory network connections can be formed and broken after a duplication event. Our quantitative approach takes the selective conditions and molecular adaptability explicitly into account, and opens up a new angle on the duplication-divergence question that is complementary to existing approaches. Evolution of network connections is treated more abstractly in numerical studies of biological network growth, which have recently received much attention [10,36,37]. The use of experimental data will help to perform such studies on a more realistic footing. Finally, the promising new field of experimental network engineering [38–40] and evolution (see e.g., [41]) will also benefit from the quantification of network adaptability.

## Materials and Methods

### Mutational dataset.

In this paper we used an extensive dataset of binding affinities of *lac* repressor and operator mutants, obtained by B. Müller-Hill and coworkers. In these experiments, repression values *F*_OiRj_ have been determined in vivo as the ratio of the unrepressed and repressed expression levels of a β-galactosidase *(lacZ)* reporter gene, controlled by a mutant *lac* operator O_i_ and mutant *lac* repressor R_j_. This was done using the standard assay by Miller [42]. Since the β-galactosidase synthesis is proportional to the fraction of free operator (see e.g., [43]), we find for the repression value *F*_OiRj_ = 1 + [R_j_]/K_D_, where K_D_ is the equilibrium dissociation constant and [R_j_] is the concentration of active repressor R_j_. The dataset contains repression values of base pair substitutions leading to changes in amino acid residues 1 and 2 of the recognition helix of the *lac* repressor (Y17 and Q18) and base pairs 4 and 5 of the symmetric *lac* operator [44]. These residues and base pairs were found to be most important for altering repressor operator-binding affinities [14]. The dataset covers a considerable fraction of all possible substitutions involving a homodimeric repressor and a symmetric operator (1,286 out of a total of 6,400). Part of this raw data is published in Lehming et al*.* [14]; the full dataset is found in [21]. The contributions of the two repressor amino acids to the repression value were found to be additive. With this knowledge, repression values could convincingly be assigned to all mutants, including those that were not measured [14]. In the present study we use these assigned repression values, all of which are given in [14]. Moreover, we extend the dataset to include heterodimeric repressors and non-palindromic operators (see below), to obtain the complete mapping between sequence and repression values for all possible mutants (1 × 10^7^) in the key repressor residues and operator base pairs.

### Repression values of heterodimers and non-palindromic operators.

We consider the repressors to act as dimers. After their duplication, once the repressors genes are mutated, this leads to heterodimerization of distinct monomers. While heterodimer binding strengths *(F_He_)* have not been directly measured, they can be derived from the two corresponding homodimer repression values *(F_Ho1_* and *F_Ho2_),* measured on a palindromic operator. The heterodimer binding energy *ΔG_He_* is the sum of the monomer-monomer and the dimer-operator binding energy. Simple equilibrium considerations lead to the following expression, where [*R*] in this case is the total concentration of repressor *subunits*:


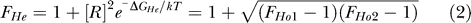


With this equation, repression values involving non-palindromic operators are also automatically taken into account: each dimer-operator interaction is built up additively [22] from two interactions between a monomer and an operator-half. In this derivation the dimerization free energy was assumed to be fixed, since it does not directly affect the specificity by which the repressors recognize their operators. The heterodimer repression value then becomes independent of the dimerization energy.

### Optimal pathway simulations.

Each repressor monomer is represented by six base pairs (two amino acid residues), and each operator by four base pairs, which are key to specific binding. The complete network with duplicates is thus represented by 20 base pairs. Each simulation run starts with the duplication of a tight binding repressor-operator pair, having a repression value of 100 or higher. Out of all possible repressor-operator combinations (homodimers and palindromic operators), there are 132 fulfilling this condition. Changing this threshold did not significantly alter the outcome of the simulations. In order to avoid any bias due to codon usage of the starting repressor, separate simulations were run starting from each of its synonymous codon versions. These simulations were averaged to produce the presented results.

In order to determine the optimal mutational pathways in the fitness landscape, an evolutionary algorithm was employed. Beginning with one of the starting sequences, each round we generated all mutants that differ by one base pair (60 in total). Of each mutant network, the strength of all eight possible interactions was determined (see Figure 1B where four possible interactions are schematically shown between the repressor dimers and one of the two operators). Interactions between repressor homodimers and palindromic operators were directly assigned from the published repression values [14]. Other interactions were calculated from the measured data as described above. Next, we selected the best *L* networks to the next round based on a fitness parameter that is directly calculated from the interaction strengths (see equation 1). The next round started by again generating all single base pair mutants of the *L* selected networks. The effect of *L* was assessed by varying it between 1 and 10^5^. Decreasing fitness steps were not allowed, and in case of equal fitness, parents were ranked above their offspring. These rules make divergence harder because they constrain the space that can be explored. The evolutionary cycle was repeated until the fitness could not be further improved. Pathways were considered to be successful when the fitness came within a factor 10 of the highest fitness in the landscape.

## Supporting Information

Figure S1 Typical Divergence Pathway, with the Additional Requirement of Continued Tight Binding of Both Repressors (*F* ≥ 100) (A) Evolving interaction network, where line thickness denotes binding strength between repressor monomer and operator-half. Dotted lines denote negligible repression. Yellow crosses indicate repressor and operator mutations, which are positioned at the top and bottom of the interaction lines respectively.
(B) Fitness trajectory (black) and corresponding repression of each repressor on its operator (red and blue). Fitness is normalized to the maximum value (~ 1 ×10^10^).
(C) Sequences for each round. Mutated positions are colored white.
(349 KB PDF)

Figure S2 Qualitative Features of Successfully Diverging Paths in the Probabilistic Pathway Simulations Simulations were performed with a 5% growth advantage of a diverged network over the initial duplicate network, and a population size of 10^5^. Of all traced paths, 17% successfully diverged, despite the strict requirements that promote trapping in local optima (fitness cannot decrease). Relaxing these conditions would lead to larger divergence probabilities.
(A) Histogram showing the number of base mutations until divergence for the successful pathways.
(B) Histogram showing the lowest repression values of each repressor on its operator during the successful divergence pathways.
(C) Histogram showing the number of neutral mutations that occur until the pathways successfully diverged.
(39 KB PDF)

Protocol S1 Simulation of Mutational Pathways Incorporating Probabilistic Population Dynamics (28 KB DOC)

Protocol S2 Comment on Neutral Mutations Required for the Emergence of a New Operator (20 KB DOC)

Protocol S3 Alternative Selective Pressures and the Escherichia coli Regulatory Network (333 KB DOC)
