# Supplementary figures and images for: Evolutionary Potential of a Duplicated Repressor-Operator Pair: Simulating Pathways Using Mutation Data
Source: PLoS Comput Biol. 2006 May 26;2(5):e58. doi: 10.1371/journal.pcbi.0020058 (PMC1464816; doi:10.1371/journal.pcbi.0020058)

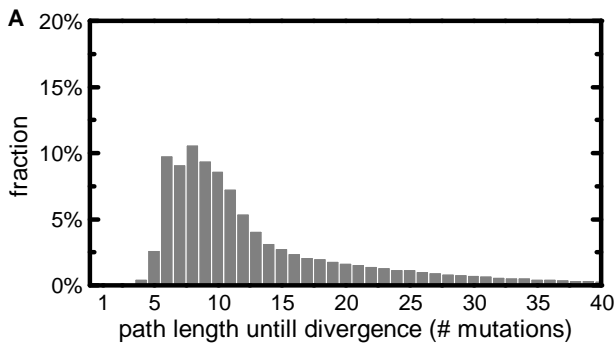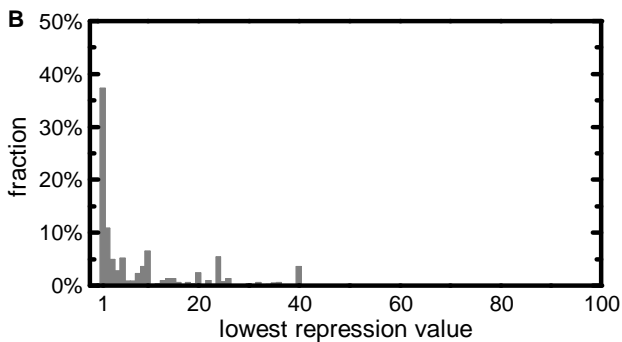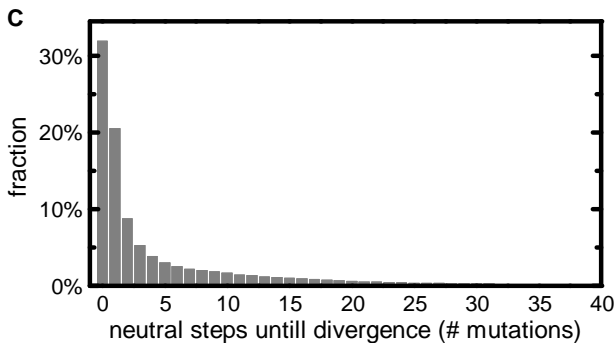

Supplement: Figure S2 — Simulations were performed with a 5% growth advantage of a diverged network over the initial duplicate network, and a population size of 105. Of all traced paths, 17% successfully diverged, despite the strict requirements that promote trapping in local optima (fitness cannot decrease). Relaxing these conditions would lead to larger divergence probabilities. (A) Histogram showing the number of base mutations until divergence for the successful pathways. (B) Histogram showing the lowest repression values of each repressor on its operator during the successful divergence pathways. (C) Histogram showing the number of neutral mutations that occur until the pathways successfully diverged. (39 KB PDF) [file pcbi.0020058.sg002.pdf]
