# Supplementary material for: Evolutionary Potential of a Duplicated Repressor-Operator Pair: Simulating Pathways Using Mutation Data
Source: PLoS Comput Biol. 2006 May 26;2(5):e58. doi: 10.1371/journal.pcbi.0020058 (PMC1464816; doi:10.1371/journal.pcbi.0020058)
Supplement: Protocol S1 — (28 KB DOC) [file pcbi.0020058.sd001.doc]

**Supporting Information Protocol S1:**

Simulation of mutational pathways incorporating probabilistic population dynamics

Here results are presented of a second simulation method, where mutations are fixed with a probability that is based on the associated fitness increase (see methods below). Compared to the optimal pathway simulations, this probabilistic approach does not search the landscape as systematically, but it is arguably closer to natural evolution, in that the fixation chance of mutations with no or lower fitness increases is more well-defined [1].

We find that the key characteristics of the probabilistic pathways are very similar to those of the optimal pathways. From every starting condition it appears possible to diverge towards independent binding while the fitness increases monotonously along the way (17% of all paths). The success rate logically differs for the different starting sequences, but all of them can yield successful trajectories. Looking at the probabilistic paths in more detail (Figure S2), we see that they are somewhat longer, but half of them still diverge within 10 mutation steps. And although the sequential network changes are not as uniform, the paths are still characterized by few neutral mutations (0 or 1 neutral steps for 50% of the paths) and an early reduction in repression of one repressor (*F*<5 for 50% of paths).

Our probabilistic model allows us to vary the amount of drift present in our pathways by varying both the effective size of a population (*N*) and the growth advantage that diverged networks have over un-diverged networks (*s*_max_). Taking a conservative growth advantage of 5% [2] we simulated probabilistic pathways for population sizes ranging from 10^3^ to 10^8^. At high population sizes pathway characteristics remain very similar. Only at population sizes below 10^4^ we start to see a strong effect of genetic drift: more neutral mutations, and hence longer pathways. However, the fraction of diverged pathways and the reduction in repression of one of the repressors does not significantly change.

In our probabilistic model we do not allow disadvantageous mutation to be fixed. An important finding we present in this paper is that for divergence to occur, fitness drops are actually not necessary. (Note that if one would allow drops in fitness, all pathways would reach divergence eventually, since trapping in local optima would then not be possible.) Even if disadvantageous mutations would be explicitly modeled, we would not expect to find other pathway characteristics for population sizes above 10^4^, as these mutations have very low fixation chances compared to the readily available beneficial mutations present in our system.

**METHODS**

***Probabilistic pathway simulations****.* To model the effect of genetic drift in the evolutionary pathways, probabilistic simulations were performed. Thousand such pathways were traced for each of the starting sequences. In each simulation step 60 different single base pair substitutions are possible (the 20 base pairs can each mutate to 3 different bases), which were assumed to occur with equal probability. The fixation probability of each specific mutation depends on its associated fitness increase, and was calculated with a standard and simple population genetics model [1, 3, see below]. In each simulation step, the fixation probabilities of all 60 possible single base pair substitutions were calculated, and one substitution was randomly chosen according to its share in the total probability. Each path was continued until a (possibly local) optimum was reached, so that the fitness could not be further improved. The purpose of this Monte Carlo-like scheme was to check whether biased random walks show similar features as the ones generated by our optimal pathway simulations.

Mutations that decrease the network fitness were assumed not to be able to fix, while those that keep the fitness constant have a fixation probability of 1/*N*, where *N* is the effective population size. Mutations that do increase the fitness have a fixation chance of 2Δ*s*, where Δ*s* is the selective advantage that this fitter mutant has over its parent. In our simulations we let an increase in the fitness parameter by a factor of 10 corresponds to a Δ*s* of 1%. In this way, a successfully diverged network has a selective advantage of 5% (*s*_max_) over the initial duplicated state (fitness rises from 10^-6^ to 10^-1^, see main text), which matches typical experimentally observed growth advantages [2, 4].

In this probabilistic scheme a mutation conferring a selective advantage Δ*s* will have *2N*Δ*s* times more chance to be accepted than a neutral mutation. Therefore, both the effective population size and the defined selective advantage influence the effectiveness of selection. By either lowering *N* or *s*_max_ the amount of genetic drift in the model increases. We typically simulated an effective population size *N*=10^5^, together with a fixed *s*_max_ = 5%. The population size needed to be lower than 10^4^ before genetic drift substantially increased the number of neutral mutations in successful paths.

**REFERENCES**

1. Kimura M (1962) On the probability of fixation of mutant genes in a population. Genetics 47: 713-719.
2. Dekel E, Alon U (2005) Optimality and evolutionary tuning of the expression level of a protein. Nature 436: 588-592.
3. MacArthur S, Brookfield JFY (2004) Expected rates and modes of evolution of enhancer sequences. Mol Biol Evol 21: 1064-1073.
4. Koch AL (1983) The protein burden of lac operon products. J Mol Evol 19: 455-462.
