# Supplementary material for: Evolutionary Potential of a Duplicated Repressor-Operator Pair: Simulating Pathways Using Mutation Data
Source: PLoS Comput Biol. 2006 May 26;2(5):e58. doi: 10.1371/journal.pcbi.0020058 (PMC1464816; doi:10.1371/journal.pcbi.0020058)
Supplement: Protocol S2 — (20 KB DOC) [file pcbi.0020058.sd002.doc]

**Supporting Information Protocol S2:**

Comment on neutral mutations required for the emergence of a new operator

Here we consider an alternative mechanism for creating a new regulatory interaction, where a new operator must emerge in an effectively random DNA sequence. In a typical prokaryotic case, like e.g. the *lac* system, a 20 base pair operator has to emerge somewhere in a roughly 100 base pair region in order to effectively block RNA polymerase binding. Then there are 10 expected prior matching base pairs for the best binding site within the promoter region. However, experimental data suggests that at least 15 base pairs need to match before appreciable binding is achieved [1, 2], from which point further mutations can be positively selected for. This means that more than 5 base pairs need to be optimized without selection, while the coevolutionary pathways can be selected for almost immediately.

**REFERENCES**

1. Betz JL, Sasmor HM, Buck F, Insley MY, Caruthers MH (1986) Base substitution mutants of the *lac* operator: in vivo and in vitro affinities for lac repressor. Gene 50: 123-132.
2. Dubertret B, Liu S, Ouyang Q, Libchaber A (2001) Dynamics of DNA-protein interaction deduced from in vitro DNA evolution. Phys Rev Lett 86: 6022-6025.
