# Supplementary material for: Evolutionary Potential of a Duplicated Repressor-Operator Pair: Simulating Pathways Using Mutation Data
Source: PLoS Comput Biol. 2006 May 26;2(5):e58. doi: 10.1371/journal.pcbi.0020058 (PMC1464816; doi:10.1371/journal.pcbi.0020058)
Supplement: Protocol S3 — (333 KB DOC) [file pcbi.0020058.sd003.doc]

**Supporting Information Protocol S3:**

Alternative selective pressures and the *Escherichia coli* regulatory network

Here we briefly comment on the possibility of alternative selective pressures after a duplication event. In the main text we have considered a selective pressure for independent regulation of two operator sites by two repressors. But other selective pressures could result in different network topologies, or motifs [1,2]. In the case of the regulators of the Lac/Gal family it is clear that cross-interactions between the operons should be eliminated: each operon should be transcribed only if the relevant carbon source is present. In other cases the cross-interactions might in fact be desirable and therefore not be selected against. For instance the so-called *bi-fan* motif (see ref [1] and Fig S3.1) might then originate from a duplication of a gene that regulated multiple genes before duplication.

In order to see whether this scenario could have materialized in the *E. coli* regulatory network, one would need to search for *bi-fan* motifs where homologous transcription factors share their operators. Interestingly, these cases are readily found. An example could be the system of repressor genes u*xuR* and e*xuR*. The UxuR/ExuR repressors are the regulators of genes involved in the transport and catabolism of fructuronate and glucuronate [3-5]. They are highly homologous (see Figs S3.3 and [6]) and indeed are very likely to bind to shared operator sites, with different affinities (see [7,8] and Fig S3.2). Moreover, they are found to be able to form heterodimers [9] and can partially substitute for one another [7,9]. As the enzymes from the UxuR/ExuR regulons have overlapping functions, there is a rationale for retaining the cross-interactions [5].

Other selective pressures for different topologies after duplication could be imagined, for example where one cross-interaction is eliminated (see fig S3.1) In the case where global regulators become duplicated, one could expect homologs to be present in the so-called Dense Overlapping Regions. Alternatively, when more than one duplication event has taken place, a ‘*tri-fan*’ (see Fig S3.1) would seem a possible outcome. Remarkably, in the *E. coli* regulatory network all of these topologies can be found, *with* the additional conditions that the transcription factors are homologous and their operators are shared.


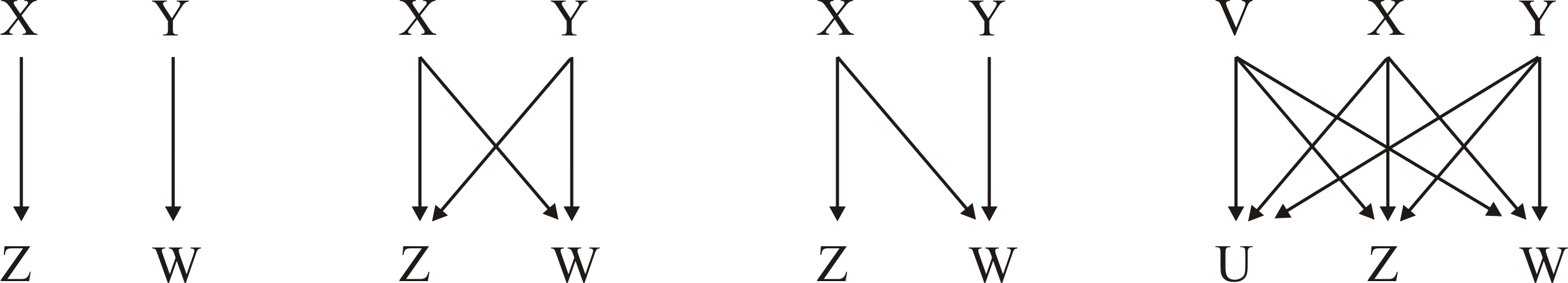


Figure S3.1 Examples of network topologies that might arise after duplication of a regulatory gene. From left to right: independent regulators, the *bi-fan*, a *bi-fan* where one connection has been lost, and a ‘*tri-fan*’.


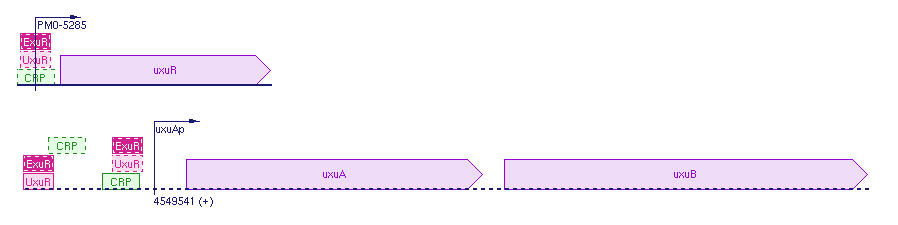


Figure S3.2: Overlapping operators and the homologous repressors ExuR and UxuR (from EcoCyc, [10]).


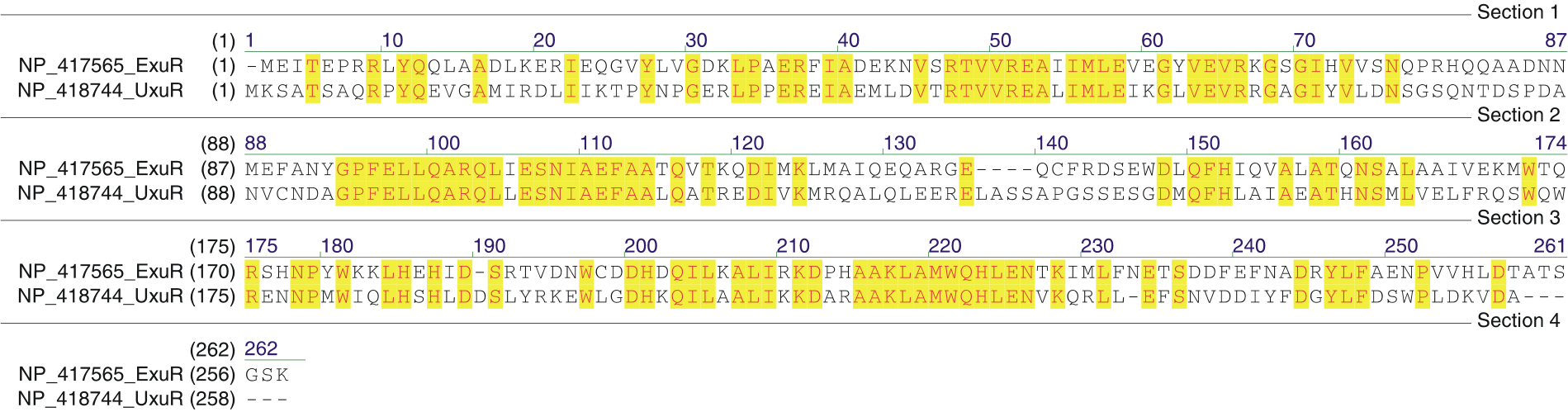


Figure S3.3: FAST alignment of the amino acid sequences of ExuR and UxuR shows 44% homology.

# REFERENCES

1. Milo R, Shen-Orr S, Itzkovitz S, Kashtan N, Chklovskii D, Alon U (2002) Network Motifs: Simple Building Blocks of Complex Networks. Science 298:824-827.
2. Shen-Orr SS, Milo R, Mangan S, Alon U. (2002) Network motifs in the transcriptional regulation network of Escherichia coli. Nat Genet 31:64-68.
3. Portalier R, Robert-Baudouy J, Stoeber F (1980) Regulation of Escherichia coli K-12 hexuronate system genes: exu regulon. J Bacteriol 143:1095-1107.
4. Ritzenthaler P, Mata-Gilsinger M, Stoeber F (1980) Construction and expression of hybrid plasmids containing Escherichia coli K-12 uxu genes. J Bacteriol 143:1116-1126.
5. Bates Utz C, Nguyen AB, Smalley DJ, Anderson AB, Conway T (2004) GntP is the Escherichia coli fructuronic acid transporter and belongs to the UxuR regulon. J Bacteriol 186:7690-7696.
6. Madan Babu M, Teichmann SA (2003) Evolution of transcription factors and the gene regulatory network in Escherichia coli. Nucl Acids Res 31: 1234-1244.
7. Ritzenthaler P, Blanco C, Mata-Gilsinger M. (1983) Interchangeability of repressors for the control of the uxu and uid operons in E. coli K12. Mol Gen Genet 191:263-270.
8. Rodionov DA, Mironov AA, Rakhmaninova AB, Gelfand MS (2000) Transcriptional regulation of transport and utilization systems for hexuronides, hexuronates and hexonates in gamma purple bacteria. Mol Microbiol 38:673-683.
9. Ritzenthaler P, Blanco C, Mata-Gilsinger M (1985) Genetic analysis of uxuR and exuR genes: evidence for ExuR and UxuR monomer repressors interactions. Mol Gen Genet 199: 507-511.
10. Keseler et al. (2005) EcoCyc: a comprehensive database resource for Escherichia coli. Nucl Acids Res 33: D334-D337.
