# Supplementary material for: Evolutionary Potential of a Duplicated Repressor-Operator Pair: Simulating Pathways Using Mutation Data
Source: PLoS Comput Biol. 2006 May 26;2(5):e58. doi: 10.1371/journal.pcbi.0020058 (PMC1464816; doi:10.1371/journal.pcbi.0020058)
Supplement: Figure S1 — (A) Evolving interaction network, where line thickness denotes binding strength between repressor monomer and operator-half. Dotted lines denote negligible repression. Yellow crosses indicate repressor and operator mutations, which are positioned at the top and bottom of the interaction lines respectively. (B) Fitness trajectory (black) and corresponding repression of each repressor on its operator (red and blue). Fitness is normalized to the maximum value (~ 1 ×10^10^). (C) Sequences for each round. Mutated positions are colored white. (349 KB PDF) [file pcbi.0020058.sg001.pdf]

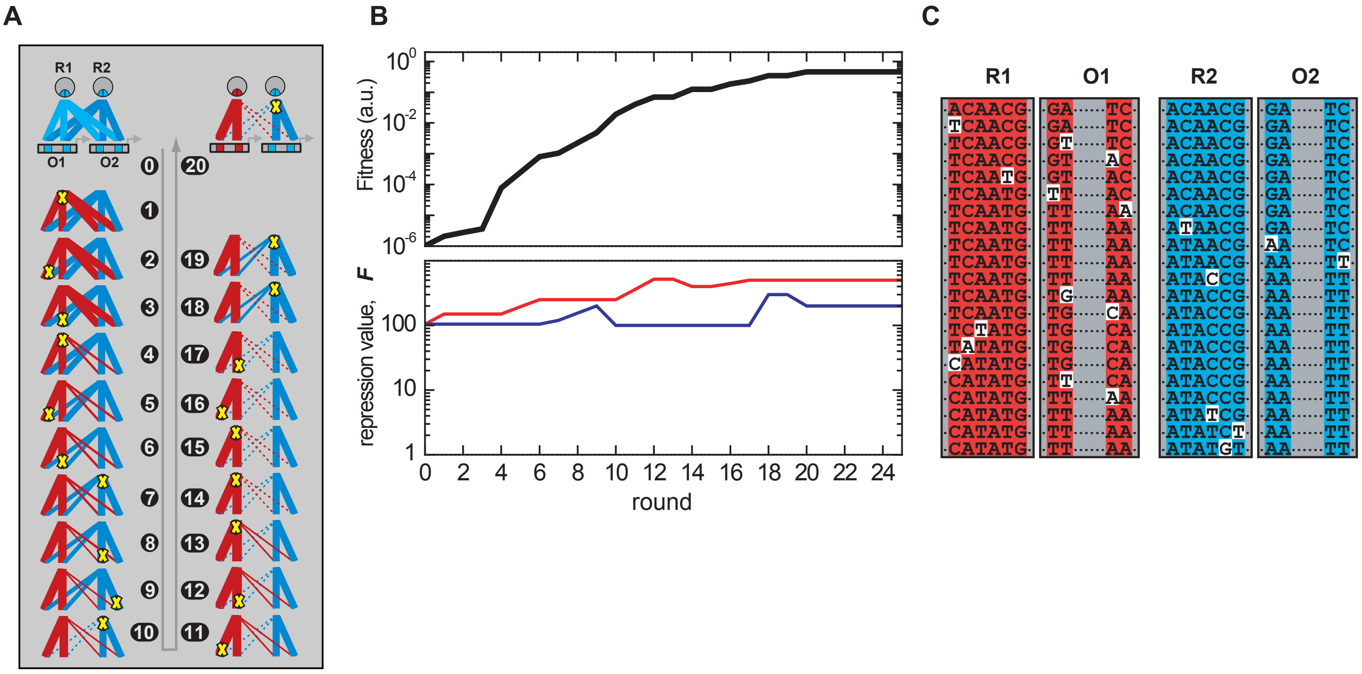
A
R1
R2
O1
O2
0 1 2 3 4 5 6 7 8 9 10 11 12 13 14 15 16 17 18 19 20
B
Fitness (a.u.)
10^{0}
10^{-2}
10^{-4}
10^{-6}
repression value, F
100
10
1
0 2 4 6 8 10 12 14 16 18 20 22 24
round
C
R1 O1 R2 O2
ACAACG GA TC ACAACG GA TC
TCAACG GA TC ACAACG GA TC
TCAACG GT TC ACAACG GA TC
TCAACG GT AC ACAACG GA TC
TCAATG GT AC ACAACG GA TC
TCAATG TT AC ACAACG GA TC
TCAATG TT AA ACAACG GA TC
TCAATG TT AA ATAACG GA TC
TCAATG TT AA ATAACG AA TC
TCAATG TT AA ATAACG AA TT
TCAATG TT AA ATACCG AA TT
TCAATG TG AA ATACCG AA TT
TCAATG TG CA ATACCG AA TT
TCTATG TG CA ATACCG AA TT
TATATG TG CA ATACCG AA TT
CATATG TG CA ATACCG AA TT
CATATG TT CA ATACCG AA TT
CATATG TT AA ATACCG AA TT
CATATG TT AA ATATCG AA TT
CATATG TT AA ATATCT AA TT
CATATG TT AA ATATGT AA TT
